# A critical assessment of SELDI-TOF-MS for biomarker discovery in serum and tissue of patients with an ovarian mass

**DOI:** 10.1186/1477-5956-10-45

**Published:** 2012-07-23

**Authors:** Wouter Wegdam, Perry D Moerland, Danielle Meijer, Shreyas M de Jong, Huub C J Hoefsloot, Gemma G Kenter, Marrije R Buist, Johannes MF G Aerts

**Affiliations:** 1 Department of Gynecology, Academic Medical Center, University of Amsterdam, Meibergdreef 9, 1105, AZ, Amsterdam, the Netherlands; 2 Bioinformatics Laboratory, Department of Clinical Epidemiology, Biostatistics and Bioinformatics, Academic Medical Center, University of Amsterdam, Meibergdreef 9, Amsterdam, AZ, 1105, the Netherlands; 3 Netherlands Bioinformatics Centre, Geert Grooteplein 28, Nijmegen, GA, 6525, the Netherlands; 4 Netherlands Proteomics Centre, H.R. Kruytgebouw, Padualaan 8, CH, Utrecht, 3584, the Netherlands; 5 Department of Medical Biochemistry, Academic Medical Center, University of Amsterdam, Meibergdreef 9, Amsterdam, AZ, 1105, the Netherlands; 6 Swammerdam Institute for Life Sciences, University of Amsterdam, 1098, XH, Amsterdam, the Netherlands

**Keywords:** Mass spectrometry, Microdissection, Ovarian cancer, SELDI, Classification, Biomarker, Serum, Tissue

## Abstract

**Background:**

Less than 25% of patients with a pelvic mass who are presented to a gynecologist will eventually be diagnosed with epithelial ovarian cancer. Since there is no reliable test to differentiate between different ovarian tumors, accurate classification could facilitate adequate referral to a gynecological oncologist, improving survival. The goal of our study was to assess the potential value of a SELDI-TOF-MS based classifier for discriminating between patients with a pelvic mass.

**Methods:**

Our study design included a well-defined patient population, stringent protocols and an independent validation cohort. We compared serum samples of 53 ovarian cancer patients, 18 patients with tumors of low malignant potential, and 57 patients with a benign ovarian tumor on different ProteinChip arrays. In addition, from a subset of 84 patients, tumor tissues were collected and microdissection was used to isolate a pure and homogenous cell population.

**Results:**

Diagonal Linear Discriminant Analysis (DLDA) and Support Vector Machine (SVM) classification on serum samples comparing cancer versus benign tumors, yielded models with a classification accuracy of 71-81% (cross-validation), and 73-81% on the independent validation set. Cancer and benign tissues could be classified with 95-99% accuracy using cross-validation. Tumors of low malignant potential showed protein expression patterns different from both benign and cancer tissues. Remarkably, none of the peaks differentially expressed in serum samples were found to be differentially expressed in the tissue lysates of those same groups.

**Conclusion:**

Although SELDI-TOF-MS can produce reliable classification results in serum samples of ovarian cancer patients, it will not be applicable in routine patient care. On the other hand, protein profiling of microdissected tumor tissue may lead to a better understanding of oncogenesis and could still be a source of new serum biomarkers leading to novel methods for differentiating between different histological subtypes.

## Background

Ovarian cancer is the leading cause of gynecologic deaths in Western countries [1]. The majority of patients are diagnosed at an advanced stage, when the 5-year survival is only 28%, compared to 95% for early-stage tumors. On the other hand, only 13-21% of patients with a pelvic mass who are presented to a gynecologist will eventually be diagnosed with epithelial ovarian cancer [2]. Furthermore, 5-10% will be diagnosed with a tumor of low malignant potential, which has a different biological behavior to that of an ovarian carcinoma. Tumors of low malignant potential also have a very low recurrence rate and a far more favorable outcome with a 5-year survival rate close to 100% in FIGO stage 1. The specific properties of these tumors allow less extensive and fertility-sparing surgery [3]. Since there is no reliable clinical test to differentiate between different ovarian tumors, the definitive diagnosis is often only obtained after surgery. Accurate initial classification of patients with an ovarian tumor could therefore prevent patients from undergoing extensive surgery in case of a benign tumor or a tumor of low malignant potential. In case of an ovarian carcinoma, accurate classification could facilitate adequate referral to a specialized hospital or gynecological oncologist improving cancer survival [4,5]. For this reason, the use of CA125 as a serum marker in combination with vaginal ultrasonography for detection of ovarian cancer has been extensively evaluated in the last decade [6]. Combining menopausal status, ultrasonographic morphology, and serum CA 125 levels in the Risk of Malignancy Index (RMI) with a cutoff level of 200 gave a sensitivity of 70.6% and a specificity of 89.3% in the ability to distinguish malignant from benign pelvic masses [7]. However, only 50% of early stage ovarian cancers are associated with an elevated level of CA125. Furthermore, CA125 is also elevated in other cancers (pancreatic, breast, bladder, liver, lung), benign disease (diverticulitis, uterine fibroids, endometriosis, ovarian cysts), and physiological conditions such as menstruation [8]. These characteristics make CA125 unreliable as a marker for detection of ovarian cancer and as a differentiating marker between different ovarian tumors [9]. When combining multiple serum tumor markers (CA125, CA72.4, and M-CSF), a higher sensitivity (70%) could be achieved compared to CA125 alone (45%), when fixing the specificity at 98%, [10]. In addition, other combinations of tumor markers like CA125, CEA, CA15-3, YKL-40 and HE4 have been studied to improve diagnosis. Studies utilizing these and other markers have shown an improved sensitivity in detecting early-stage disease and discriminating between different pelvic masses [11,12]. However, the only single biomarker for ovarian cancer which was recently approved by the Food and Drug Administration is HE4. It was approved for monitoring recurrence but not for discriminating between different ovarian tumors. The OVA1 test, FDA approved in 2009, is a multimarker diagnostic test combining the expression of five proteins (CA125, transthyretin, apolipoprotein A1, beta 2 microglobulin, and transferrin) complementing the physician’s preoperative assessment [13]. However, these and other proteins did not improve sensitivity for preclinical diagnosis beyond CA125 alone and should therefore not be used for screening purposes [14].

With the introduction of high-throughput mass spectrometry techniques such as SELDI-TOF, the search for biomarkers which could enhance the discriminatory accuracy of existing tumor markers was fueled [15,16]. The above mentioned OVA1 test originated from such discovery work using SELDI-TOF profiling [17]. Despite the initial promises of this approach, no new biomarkers detected by mass spectrometry are in clinical use today. This can be largely attributed to shortcomings in experimental protocols concerning sample collection, sample storage, and bioinformatic analysis of the data generated in SELDI studies [18]. Moreover, many of the earlier SELDI studies lack external validation [15,19]. Recent studies using SELDI-TOF-MS have taken into account these limitations and have taken precautionary steps towards robust identification and validation of the profiles identified [20-22]. Concerns about reproducibility of SELDI-TOF generated data have also been addressed in recent papers with experimental methods being applicable to other forms of mass-spectrometry [23]. Together these results have shown that SELDI-TOF can be used to search for differentially expressed proteins within a large group of samples in a relatively short time.

In this study, we aimed to incorporate the essential aspects of a classification study to evaluate the suitability of SELDI-TOF in generating a reliable and reproducible biomarker profile in serum and tissue to differentiate between different ovarian tumors. Instead of healthy women, patients with a benign ovarian tumor were chosen as control group. Patients with a tumor of low malignant potential were also included as a separate group because of their different biological and clinical behavior. Comparison of these patient groups enables the identification of protein profiles that differentiate between various tumors of the ovary. Prospectively collected samples, strict protocols, and extensive use of technical replicates for quality control, combined with an independent validation cohort in the serum experiments were used to obtain a robust and reliable outcome.

In addition to the serum profiling we used SELDI-TOF for protein profiling directly in laser-microdissected tumor-tissue lysates from a subset of the same patients. The use of dissected homogeneous tumor cell populations leads to more reproducible protein patterns without the problems associated with serum, such as its huge dynamic range. As a result we expect that using tissue samples more accurate classification models can be generated for differentiating between tumor types. By profiling and comparing tumor tissue with serum we aim to identify peaks found in both serum and tissue resulting in the identification of reliable tumor produced serum biomarkers. A clinical serum-based assay using a tumor-specific biomarker panel could assist the clinician in differentiating between different tumor types, without the need for a tissue biopsy, subsequently facilitating adequate treatment selection or referral to a gynecological oncologist.

## Results

### Differential expression in serum samples

Pairwise comparison of serum spectra from patients with ovarian cancer and benign tumors identified 145 differentially expressed peaks for the CM10, out of a total of 265 peaks. 71 peaks out of 156 peaks were differentially expressed for the Q10 data (adjusted p-value < 0.05). Analysis of serum from cancer patients versus serum of patients with a tumor of low malignant potential yielded 38 differentially expressed peaks for CM10 and 13 peaks for Q10. No differentially expressed peaks were found when comparing the serum of patients with a benign tumor and the serum of patients with a tumor of low malignant potential. The ten most differentially expressed peaks between patients with ovarian cancer and a benign tumor, their adjusted p-values, and their fold changes are shown in Table 1. Figures with examples of the spectra obtained for the different conditions and chip types can be found in the supplementary information (see Additional file 1) We also added an example of a peak differentially expressed in serum on the CM10 array between a patient with a benign and malignant ovarian mass (see Additional file 2).

**Table 1:** Comparison of detected peaks in serum and tissue

	**Serum**			**Tissue**		
	**m/z**	**adjusted p-value**	**FC**	**m/z**	**adjusted p-value**	**FC**
CM10	10844	<0.0001	2.35	11753	<0.0001	4.11
	2773	<0.0001	0.57	4309	<0.0001	0.09
	3784	<0.0001	0.46	11855	<0.0001	3.48
	5963	<0.0001	1.76	12294	<0.0001	4.84
	2719	<0.0001	0.61	6652	<0.0001	2.39
	6048	<0.0001	1.60	6987	<0.0001	2.16
	6081	<0.0001	1.61	3139	<0.0001	0.21
	2796	<0.0001	0.58	9983	<0.0001	2.00
	5820	<0.0001	1.77	24815	<0.0001	1.99
	4296	<0.0001	0.51	5768	<0.0001	0.31
Q10	6685	<0.0001	0.64	3783	<0.0001	12.23
	3694	<0.0001	0.62	2774	<0.0001	0.11
	4288	<0.0001	0.70	4219	<0.0001	0.31
	4685	<0.0001	1.31	3958	<0.0001	2.85
	4796	0.0001	1.64	2923	<0.0001	2.85
	2909	0.0002	0.68	5460	<0.0001	0.19
	2858	0.0002	0.70	2944	0.0001	1.99
	1910	0.0005	0.69	4298	0.0007	0.36
	7994	0.0005	1.54	8198	0.0007	1.99
	4631	0.0012	1.24	5198	0.0009	1.80

List of ten m/z values most differentially expressed when comparing cancer with benign tumors in the serum and tissue experiments. Peaks listed are after pre-processing for CM10 and Q10 arrays. For each of the m/z values the adjusted p-value and fold change (FC: cancer versus benign) is given.

### Classification and validation of serum samples

The average classification accuracy for discriminating ovarian cancer from benign tumors in serum samples was 79-81% for the CM10 training cohort, with a sensitivity of 75% and a specificity of 83-86% using DLDA and SVM classifiers (Table 2). When classification was performed on the Q10 dataset, the percentage of accurately classified samples was 71-73%, with a sensitivity of 69-71% and a specificity of 73-75%. Classification on CM10 data outperforms classification on Q10 data which is in agreement with the lower number of differentially expressed peaks detected for Q10. On the combined CM10/Q10 dataset the average classification accuracy was 79-81% with a sensitivity of 74-75% and a specificity of 82-87%. Results illustrate that classification performance is quite reproducible across different classifiers and types of arrays.

**Table 2:** Classification results

**A**						
**Serum training cohort**						
	CM10			Q10		
	accuracy	sens	spec	accuracy	sens	spec
DLDA	81	75	86	73	71	75
SVM	79	75	83	71	69	73
**Serum validation cohort**						
DLDA				73	88	62
SVM				81	81	81
**B**						
**Tissue predicted** (%)						
	Benign		LMP		Cancer	
Benign (true)	82.1		17.6		0.3	
LMP (true)	10.6		64.5		24.9	
Cancer (true)	3.4		10.6		86.0	

**A**. Classification of serum samples on the training and independent validation cohort. Training cohort: average classification accuracy, sensitivity (sens), and specificity (spec) (in percentage) of discriminating ovarian cancer versus benign tumor for CM10 and Q10 arrays on 500 test sets (repeated random sampling; size training sets: 80, size test sets: 47 for CM10, 48 for Q10). Validation cohort: classification accuracy, sensitivity, and specificity (in percentage) of the classifiers trained on all serum training data for Q10 validation data only. Classification models: SVM (support vector machine) and DLDA (diagonal linear discriminant analysis) with feature selection.

**B.** Confusion matrix giving the percentage of cases (average over 500 test sets) from one class classified into each of the three classes. Rows correspond to the correct class, columns to the predicted class. Results are for DLDA with three different classes: benign tumors, tumors of low malignant potential (LMP) and cancer tissue (Q10 data).

**Figure 1:**
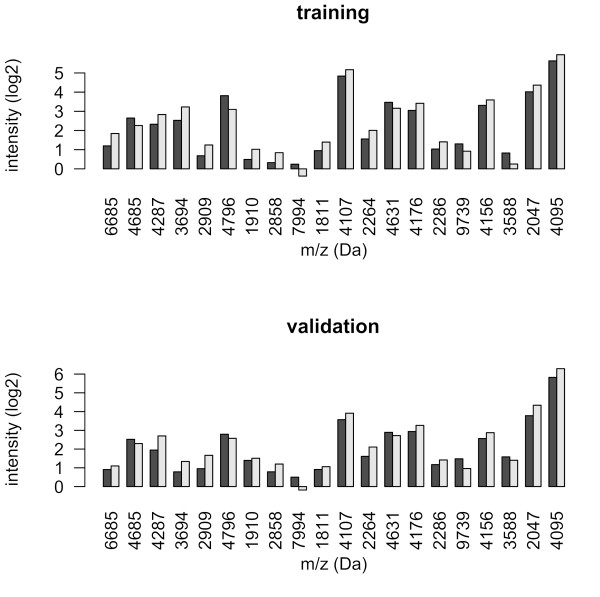
**Differentially expressed peaks included in the classification model.** Barplot of the average expression intensity (log2-scale) in the training and validation cohort for the 20 peaks included in the SVM classifier (Q10 data). Black bars represent cancer, gray bars benign tumor.

Classification analysis was also performed including patients with a tumor of low malignant potential as a third class. Classification results were inferior to the results obtained when comparing cancer and benign tumors. Average accuracy reached only 50-64% across different classifiers and ionization surfaces, versus an accuracy of 71–81% when comparing the two groups. The predictive accuracy of the resulting two-class classifiers was assessed on an independent validation set measured on Q10 arrays. The spectra of the independent validation set showed good reproducibility. Analyses using DLDA and SVM classifiers resulted in a classification accuracy of 73-81% with a sensitivity of 81-88% and specificity of 62-81% for the Q10 validation data (Table 2). Differentially expressed peaks included in the classification model estimated on the training cohort were similarly up or down regulated in the validation cohort (Figure 1).

### Differential expression in tissue samples

**Figure 2:**
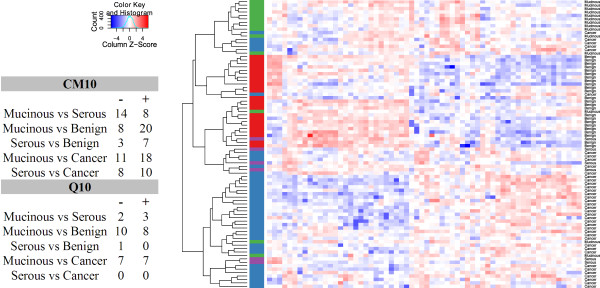
**Pairwise comparisons between tumors of low malignant potential and hierarchical clustering of tissue data.** Table with pairwise comparisons between mucinous and serous tumors of low malignant potential, cancer and benign tumor tissue. The number of upregulated (+) and downregulated (−) peaks is given for each comparison. Hierarchical clustering of CM10 tissue data. Clustering of 84 tissue samples using complete linkage and Pearson correlation distance on the 62 peaks that were differentially expressed (adjusted p-value < 0.05) between cancer, low malignant potential (divided in mucinous and serous), and benign tumor. The Z-score is calculated on the columns by subtracting the mean expression value of a column from each of the values and then dividing the resulting values by the standard deviation of the column. Color in the heat maps, therefore, indicates the relative expression level, with red being higher and blue lower than the mean expression value. The vertical side bar indicates the sample type: benign (red), mucinous LMP (green), serous LMP (purple), cancer (blue).

Pairwise comparisons between the three patient groups in microdissected tissue samples showed that not only could cancer tissue be distinguished from ovarian epithelial cells derived from benign ovarian tumors, but that tumors of low malignant potential could be distinguished from cancer and benign tissue as well. There were 68 differentially expressed peaks, out of a total of 115, when comparing cancer with benign tissue for CM10 and 42 peaks, out of a 145 peaks, for the Q10 data. Comparing tissue from tumors of low malignant potential with cancer yielded 32 and 14 differentially expressed peaks respectively. Comparison of tissue from tumors of low malignant potential and benign tissue yielded 23 differentially expressed peaks for CM10 and 13 peaks for Q10. Pairwise comparisons between all patient groups while subclassifying the low malignant potential group into mucinous and serous tumors (Figure 2) indicated that the expression profiles of serous and mucinous tumors of low malignant potential are different. When performing cluster analysis serous tumors of low malignant potential could not be identified as a separate group (Figure 2). These results suggest that mucinous tumors of LMP and, to a lesser extent, serous tumors of LMP can be identified as separate subtypes based on their protein profiles in tissue.

Measurements in serum and tissue from the same patients enabled us to compare protein spectra and to look for similarities between serum and tissue. However, there were no corresponding peaks between serum and tissue lysates (Table 1).

### Classification of tissue samples

Classification was performed as described for the serum experiments using a double cross-validation scheme. DLDA on samples of epithelial tissue from benign ovarian tumors versus tissue from ovarian cancer resulted in a model with a classification accuracy of 99% on the CM10 dataset, with a sensitivity of 99% and a specificity of 100%. For the Q10 dataset the classification accuracy of DLDA was 95%, with a sensitivity and specificity of 95%. On the combined CM10/Q10 dataset the classification accuracy of DLDA was 99% with a sensitivity of 98% and a specificity of 100%. When including tumors of low malignant potential as a third class, the accuracy dropped to 82% (CM10), 80% (Q10), and 84% (CM10/Q10) respectively. The confusion matrix for DLDA on the Q10 data (Table 2) gives the predictive accuracy for each of the three classes (benign, low malignant potential, and cancer). Tumors of low malignant potential were classified as benign in 10.6% and as cancer in 24.9% of the cases.

## Discussion

With this study, we are one of the first to describe a SELDI-TOF MS study on ovarian cancer patients in which the essential aspects of a properly designed mass spectrometry-based classification study are incorporated. We used strict protocols and a well-defined patient population in combination with an independent group of patients for validation of the classification results. Nevertheless, reliable results can only be achieved when careful patient selection is combined with stringent sampling protocols and sound pre-processing and classification. When working with large mass spectrometry generated datasets, performing multiple quality control checks during the processing of the data is of vital importance. In our study, quality control consisted of visual inspection of the spectra, assessment of reproducibility based on technical replicates, and hierarchical clustering of the pre-processed data. Several unreliable samples were detected, which were disregarded for further analyses.

Previous studies based their classification results on comparing patients with advanced ovarian cancer and healthy control patients [16,24]. These and other studies have found a number of proteins associated with ovarian cancer [15,25]. Most of these proteins however, are acute-phase reactants and as such epiphenomena most likely not specific for ovarian cancer. In the current study, we compared samples from ovarian cancer patients with samples from patients with a benign tumor of the ovaries or an ovarian tumor of low malignant potential. Therefore, the peaks included in the classification model are more likely to correspond to proteins that are specific for different types of ovarian tumor instead of reflecting a more general response to disease. The fact that our control group consisted of benign tumors might also explain why differentially expressed peaks in our study do not match the peaks found in other studies with less well defined or completely healthy control groups.

The careful selection of patient and control groups was also done to see if a model could be developed best suited for classification of patients visiting a gynecologist and diagnosed with a tumor of the ovaries rather than to create a model for population-wide screening. When comparing serum from patients with a benign tumor with serum from ovarian cancer patients, we were able to achieve a sensitivity of 69% and specificity of 73% using SVM on the Q10 training cohort, as evaluated using cross-validation. However, the utility of a classification model should always be established in an independent validation cohort [26]. In this study, we validated the model on a new group of patients and we showed that using a SVM a sensitivity of 81% and specificity of 81% could be achieved for the Q10 dataset, illustrating the robustness of our results.

However, our classification results do not outperform already existing diagnostic methods like the Risk of Malignancy Index of ovarian tumors using the serum marker CA125 and ultrasonography. Results from the UKCTOCKS study showed a sensitivity of 89.4% and a specificity of 99.8% for all primary ovarian and tubal cancers [6]. In our patient population, serum CA125, alone has a sensitivity of 81% in the total training set and of 75% in the validation set at a fixed specificity of 98%. The sensitivity in the validation set of our SVM model is only 44% at a specificity of 98%.

When including tumors of low malignant potential as a third class in the classification model, the classification results showed a poor accuracy of 50-64%. This is probably due to serum samples of patients with a tumor of low malignant potential having a high resemblance to serum samples of patients with a benign tumor, as witnessed by the absence of differentially expressed peaks. This in turn could be explained by the fact that tumors of low malignant potential not only have a histological resemblance to benign tumors but also probably do not cause acute phase responses to the same extent as a large infiltrative tumor does. In summary, these results show that SELDI-TOF generated data are not suitable for accurate classification of patients with a tumor of low malignant potential as a separate subgroup, based on their serum samples.

Besides determining serum protein profiles, we also investigated the protein profiles of the actual tumors in 84 of those patients. We obtained homogenous cell populations using laser microdissection. Measurements in tumor tissues circumvent the limitations associated with serum protein profiling, such as the dominance of highly abundant proteins and large inter-individual differences [27]. On tumor tissue samples our classification model achieved a classification accuracy between 95-99% with a sensitivity between 95-99% and a specificity between 95-100% for the different classification models and chip types. Even when tumor tissue of low malignant potential was included in the model as a third class, a classification accuracy of 80-82% was obtained. Using cluster analysis we showed that the different histological subtypes of tumors of low malignant potential can be detected using SELDI-TOF generated data from tissue lysates (see Figure 2.).

Remarkably, none of the peaks differentially expressed between the serum samples of the three patient groups (benign, low malignant potential, and cancer) were found to be differentially expressed between the tissue lysates of those same groups. This might be due to the fact that the proteins differentially expressed in tissue lysates are intracellular proteins which do not enter the bloodstream, or are present at very low concentrations making them difficult to detect. SELDI-TOF may not be sensitive enough to detect tiny amounts of tumor derived peptides in serum which has a huge dynamic range. The use of pre-fractionation methods in combination with mass-spectrometry techniques could be used to see whether tumor derived proteins, present in very small concentrations could be picked up. The lack of common peaks in de protein spectra of serum and tissue of the same patients also reveals that the proteins differentially expressed in serum of the different groups are not tumor produced proteins but epiphenomena associated with the different tumor groups.

## Conclusion

Despite the initial promise of high throughput mass spectrometry techniques such as SELDI-TOF, there are a number of limitations which have only recently been addressed [18,28]. Our study shows that with careful study design, sound processing of the data, and independent validation reliable classification results and new leads in tumor etiology can be obtained.

However, our data also show that SELDI-TOF-generated serum profiles are not able to outperform existing clinical methods such as the combination of CA125 and ultrasonography when it comes to differentiating between patients with an ovarian tumor. When searching for tumor-derived proteins in serum by comparing the serum and tissue profiles no tissue-derived proteins could be detected in serum via SELDI-TOF. However, the differentially expressed proteins found in tissue lysates could still lead to a better understanding of oncogenesis and be a source of new biomarkers when detected in serum by other more sensitive techniques. This could result in a clinical serum-based assay using a tumor-specific biomarker panel.

Although we believe that high-throughput methods like SELDI-TOF can still be put to good use when looking for differences or similarities in large patient populations, novel methods in mass spectrometry such as qualitative and quantitative proteomic analysis by nanoscale LC-MS (Liquid Chromatography-Mass Spectrometry) are being used more frequently [29]. These methods enable researchers to directly identify and quantify the proteins in a particular sample and circumvent the difficult and time-consuming process of identifying individual proteins of interest found in SELDI-TOF experiments. Although these methods offer advantages over SELDI-TOF, they are not yet suited for the large numbers of samples that SELDI-TOF can handle.

## Methods

### Samples

After informed consent was obtained, samples were prospectively collected from newly diagnosed patients at the Academic Medical Center. The study was approved by the Ethical Committee at the Academic Medical Center. Both training and validation cohort consisted of women with non-familial epithelial ovarian cancer and patients with tumors of low malignant potential. The control group consisted of patients with a range of benign ovarian tumors such as serous and mucinous cystadenoma, mature cystic teratoma, or fibroma of the ovary. Patients were matched for age, BMI, and sample storage time (Table 3). The validation cohort was collected as a separate group, after inclusion of the patients participating in the first experiment.

**Table 3:** Patients characteristics

	**Serum (training cohort)**			**Serum (validation cohort)**			**Tissue**		
	**Cancer**	**LMP**	**Benign**	**Cancer**	**LMP**	**Benign**	**Cancer**	**LMP**	**Benign**
Patients	53	18	57	16	5	21	40	20	24
Age (years)	59.2 (11.4)	53.5 (10.3)	56.6 (12.4)	58.0 (14.8)	43.3 (8.0)	54.0 (14.5)	55.8 (11.7)	50.7 (10.3)	53.4 (15.3)
BMI	26.1 (5.4)	28.7 (8.0)	26.9 (6.0)	26.5 (4.5)	27.4 (5.8)	26.6 (4.1)	26.1 (4.5)	28.8 (8.0)	26.7 (5.0)
Premenopausal	10	6	22	5	5	11	13	10	9
Postmenopausal	43	12	35	11		10	27	10	15
Sample age (years)	2.5 (0.9)	2.3 (0.5)	2.5 (0.6)	0.7 (0.4)	0.6 (0.2)	1.7 (1.2)	2.5 (1.2)	2.5 (1.0)	2.7 (0.9)
CA125 kU/L	2451	63	42	5078	131	67	4117	89	26
(range)	(7–14100)	(6–396)	(1–164)	(111–67448)	(56–284)	(2–312)	(7–67448)	(6–396)	(5–164)
**Histological type**									
Serous	38	13		13	3		32	6	
Mucinous	5	5		1	2		2	14	
Endometrioid	3			1			2		
Undifferentiated	5			1			3		
Clearcell	2						1		
**Grade**									
1	2						2		
2	12			3			9		
3	39			12			29		
NA				1					
**Figo Stage**									
I	6						6		
II	3						1		
III	36			13			27		
IV	8			3			6		

Clinical and pathological characteristics of the three separate patient groups: cancer, low malignant potential (LMP) and benign tumor. The characteristics are given for the serum training, serum validation and tissue experiments. Age, body mass index (BMI) and sample age are given as a mean value with their standard deviation. In one patient differentiation grade was not available (NA).

Clinical and pathological characteristics of the three separate patient groups: cancer, low malignant potential (LMP) and benign tumor. The characteristics are given for the serum training, serum validation and tissue experiments. Age, body mass index (BMI) and sample age are given as a mean value with their standard deviation. In one patient differentiation grade was not available (NA).

Serum samples were collected before treatment using a strict protocol. Blood was collected by the same operator at least two hours after the patient’s last meal and left to clot for 30 minutes. After centrifugation (at 1750 x g) serum was immediately frozen and stored at −80°C. Samples used were only thawed once.

From 84 patients included in the serum experiments, tissue samples were available (Table 3). Histological analysis was carried out by 2 gynecological pathologists. Tissue was snap frozen in liquid nitrogen and stored at −80°C within 30 minutes of surgery. 10 μm cryostat sections stained with hematoxylin were prepared in order to detect tissue areas of interest for microdissection. Corresponding consecutive unstained tissue sections were mounted on a microscope slide coated with a membrane (polyethylene naphtalate (PEN) Zeiss/Palm, Bernried, Germany) and stored at −80°C. Tissue areas were cut using a Veritas™ Microdissection System (Arcturus Molecular Devices, CA, USA). Using microdissection we obtained samples containing at least 90% cancer cells or tumor cells of low malignant potential, and 60% epithelial cells derived from benign ovarian tumors. Tests were performed to determine optimal lysis conditions and number of cells necessary for SELDI-TOF analysis. A tissue sample of 45.000 cells giving a protein yield of 250 ng per 1000 cells, as determined by a Lowry protein measurement, gave a reliable protein profile. Cells were denatured in 20 μl 0.1% RapiGest detergent solution (Waters Corp., Milford, MA) and heated at 80°C for 15 minutes. After centrifugation at 1750 x g for 10 min, supernatants were collected.

### SELDI-TOF MS

Protein profiles of serum and tissue samples were generated using anionic surfaces of CM10 and cationic surfaces of Q10 ProteinChip arrays (Ciphergen Biosystems Inc., Fremont, CA.). Protocols were performed as described previously [22]. In order to avoid confounding of the effect of interest (patient status) with between-chip effects, allocation of samples to each array was stratified with respect to patient status. To assess intra-chip reproducibility, randomly selected samples were duplicated on half of the arrays. To assess inter-chip reproducibility, three randomly selected serum samples from the serum training cohort, one from the validation cohort, and two tissue samples were put on three different chips. The arrays were read on a PBSII reader (Ciphergen Biosystems) with a laser intensity of 175, a detector sensitivity of 7, and a detection size range between 1.5 and 20 kDa. Calibration was performed once before measuring the arrays in rapid succession. The validation cohort was measured on Q10 ProteinChip arrays 6 months after the measurements on the training cohort.

### Pre-processing and peak detection using Ciphergen ProteinChip Software

Pre-processing was done with commercial ProteinChip Software (version 3.1.1, Ciphergen Biosystems) as described earlier [22]. In summary, spectra were normalized to the average total ion current in the mass range from 2–20 kDa (CM10) and 1.7 - 20 kDa (Q10) in the serum experiments and from 2.5 - 50 kDa (CM10) and 2–50 kDa (Q10) in the tissue experiments. Optimal settings for peak detection were chosen by visual inspection of the detected peaks (data not shown). In data obtained from serum samples, the values for the peak detection parameters (i) First Pass (signal-to-noise ratio (S/N)), (ii) Min Peak Threshold (% of all spectra), (iii) Cluster Mass Window (% of mass), and (iv) Second Pass (S/N) were set at 5, 25, 0.3 and 2. For pre-processing of the validation set, calibration coefficients and an external normalization coefficient were estimated on the training cohort only. Peak clusters were loaded from the training cohort, avoiding an information leak when pre-processing the validation data [22]. Pre-processing of the data from the tissue experiments was done using the settings 5, 5, 0.3 and 2 for the four peak detection parameters. Peak intensities that were zero or negative after baseline correction were set equal to half the minimum of the positive corrected intensities for that peak. Furthermore all peak intensities were log2-transformed in order to stabilize their variance. For both serum and tissue we also made a combined CM10/Q10 dataset by taking the union of the CM10 and Q10 peak intensities for each sample.

To see whether a different pre-processing method would achieve similar results, we also pre-processed the serum samples using the mean spectrum technique from the publicly available Cromwell software developed by the bioinformatics group at the MD Anderson Cancer Center [30]. Pre-processing results were in agreement with those of the Ciphergen ProteinChip software (data not shown). A detailed comparison between the different pre-processing methods can be found in our previous study [22].

### Quality control

Quality control consisted of (*i*) visual inspection of the spectra, (*ii*) assessment of intra- and inter-chip reproducibility based on technical replicates, (*iii*) hierarchical clustering of the pre-processed data. Based on visual inspection, seven tissue samples on one of the Q10 arrays were left out, since their spectra had very high and irregular baseline levels. Hierarchical clustering identified one serum training sample on a CM10 array as a potential outlier. This sample was discarded and pre-processing was repeated on the remaining samples.

### Statistical analysis

Pre-processed data of technical replicates were averaged. In order to detect any chip biases, we performed a factorial analysis of variance (ANOVA) with patient status and chip as main effects. The ANOVA analysis showed clear evidence of a chip effect for a number of peaks on the Q10 arrays for both serum and tissue samples. However, no significant interaction effect between patient status and chip was detected. None of the peaks that could differentiate between chips could also differentiate between patient groups (data not shown). A moderated t-test was then used to identify peaks that were pairwise differentially expressed between patient groups. Resulting p-values were corrected for multiple testing using the Benjamini-Hochberg False Discovery Rate (FDR) adjustment. Tests were considered significant if the adjusted p-values were <0.05.

### Classification

To test whether patient status could be predicted from the peak profiles obtained, we used two classification models often used for microarray and proteomics data, linear Support Vector Machines (SVM) and Diagonal Linear Discriminant Analysis (DLDA) [31]. These two models were selected because they were among the top performers in our previous study where multiple linear and non-linear classification models were compared on clinical SELDI-TOF datasets [22]. Classification analysis was performed for the discrimination of cancer from benign tumors (2-class) and when including tumors of low malignant potential as a third class. Model training and evaluation was performed as described previously [22]. In short, models were validated with repeated random sampling methodology, as advocated by Michiels *et al.*[32]. Random splits of each dataset of *N* samples were performed to generate 500 different training sets (size *n*) and associated test sets (size *N–n*). In each of the random splits, the number of samples for the patient groups was balanced in both the training and test sets. The accuracy of the resulting classifiers was assessed on the corresponding test sets. To investigate the influence of the training set size on the accuracy of the classifiers, we varied the training set size (*n* = 40,60,80 for the serum dataset and *n* = 20,40,60 (three-class) or *n* = 20,35,50 (two-class) for the tissue dataset. If not mentioned otherwise, results reported are for the largest training set size. The optimal values for the cost hyperparameter of the SVMs were estimated using 5-fold cross-validation on the training set. Feature selection was used to extract the peaks most informative for predicting patient status. For each training set, an optimal classifier was identified from the 5, 10,…,50 peaks with the highest correlation between expression and patient status as determined by either t-statistics (two-class) or F-statistics (three-class). The optimal number of peaks was again selected with 5-fold cross-validation on the training set. Such a double cross-validation scheme provides an almost unbiased estimate of the true error [33].

In the serum experiments an independent group of validation samples was available. These samples were classified by models trained on the serum training cohort only and with optimal values for the hyperparameters (# features, SVM cost parameter), as estimated on the training cohort by the double cross-validation scheme described above. Statistical analyses were performed using Bioconductor packages and in-house scripts in the statistical software package R [34].

## Abbreviations

ANOVA: Analysis of variance; BMI: Body mass index; DLDA: Diagonal linear discriminant analysis; FDR: False discovery rate; FIGO: Fédération Internationale de Gynécologie Obstétrique; LCM: Laser capture microdissection; SVM: Support vector machine.

## Competing interests

The authors declared no conflict of interests.

## Authors’ contributions

WW collected samples and clinical information, carried out the SELDI-TOF experiments, interpreted results and drafted the manuscript. PDM carried out the statistical analyses, interpreted the results and drafted the manuscript. DM interpreted the results and critically read the manuscript. SJ carried out the SELDI-TOF experiments and interpreted the results. HCJH advised on statistical analyses and interpretation of results. GGK interpreted the results. MRB was responsible for the design of the study and sample collection. JMA contributed materials and was responsible for the design of the study. All authors read and approved the final manuscript.

## Supplementary Material

Additional file 1 **Representative SELDI spectra.** Spectra (x-axis: m/z ratio (Da); y-axis: normalized intensity) obtained for the different chip types (CM10 and Q10) and specimens (serum and tissue) from the same patient with a serous adenocarcinoma. Click here for file

Additional file 2 **Representative SELDI spectra.** Spectra (x-axis: m/z ratio (Da); y-axis: normalized intensity) showing a differentially expressed peak at 10,884 Da (see Table 1 in the main text) between a patient with a benign and a patient with a malignant ovarian mass. The spectra were obtained using serum on a CM10 array. Click here for file
