# Supplementary figures and images for: A critical assessment of SELDI-TOF-MS for biomarker discovery in serum and tissue of patients with an ovarian mass
Source: Proteome Sci. 2012 Jul 23;10:45. doi: 10.1186/1477-5956-10-45 (PMC3494530; doi:10.1186/1477-5956-10-45)

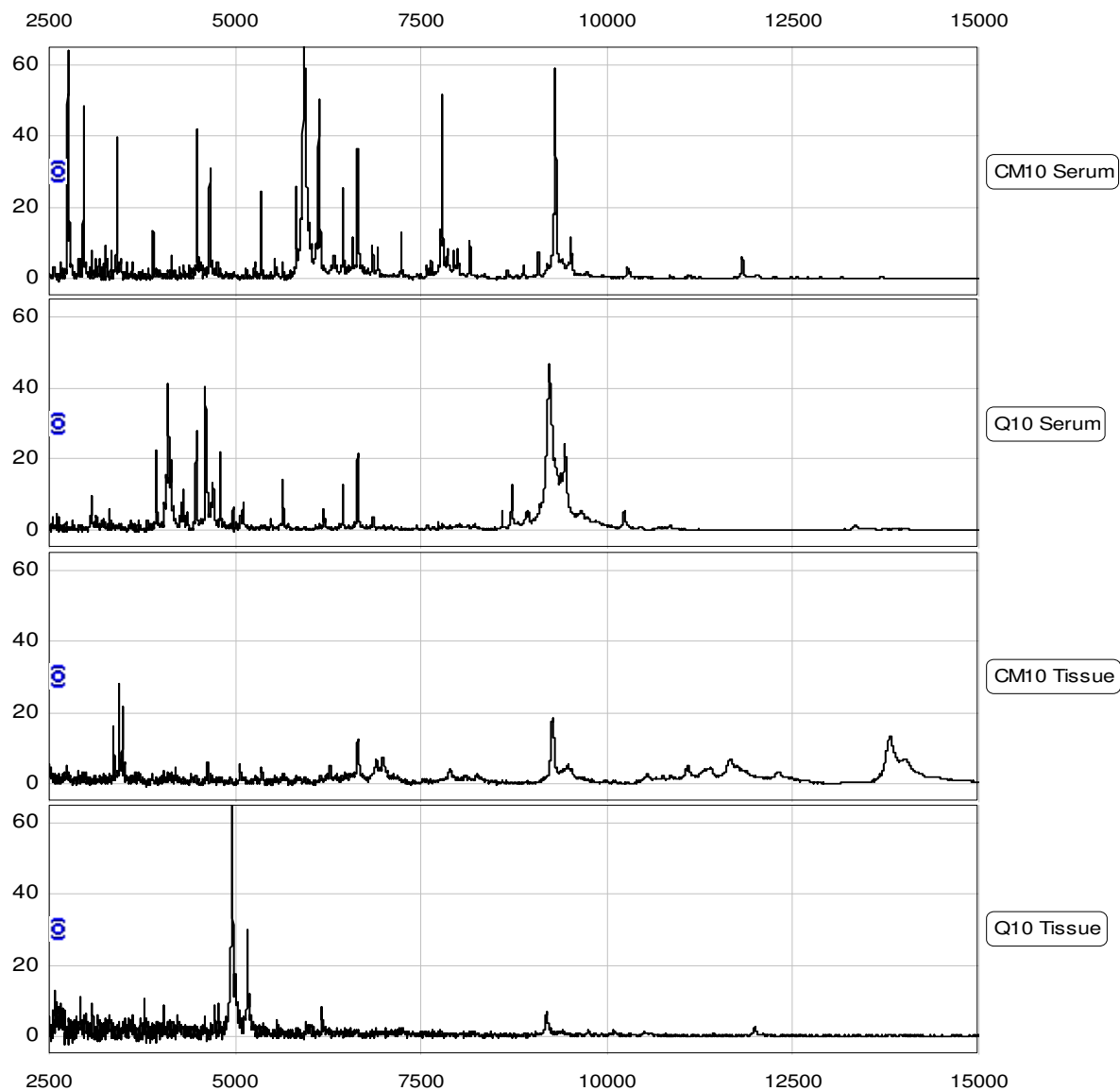

Supplement: Additional file 1 — Representative SELDI spectra. Spectra (x-axis: m/z ratio (Da); y-axis: normalized intensity) obtained for the different chip types (CM10 and Q10) and specimens (serum and tissue) from the same patient with a serous adenocarcinoma. [file 1477-5956-10-45-S1.pdf]

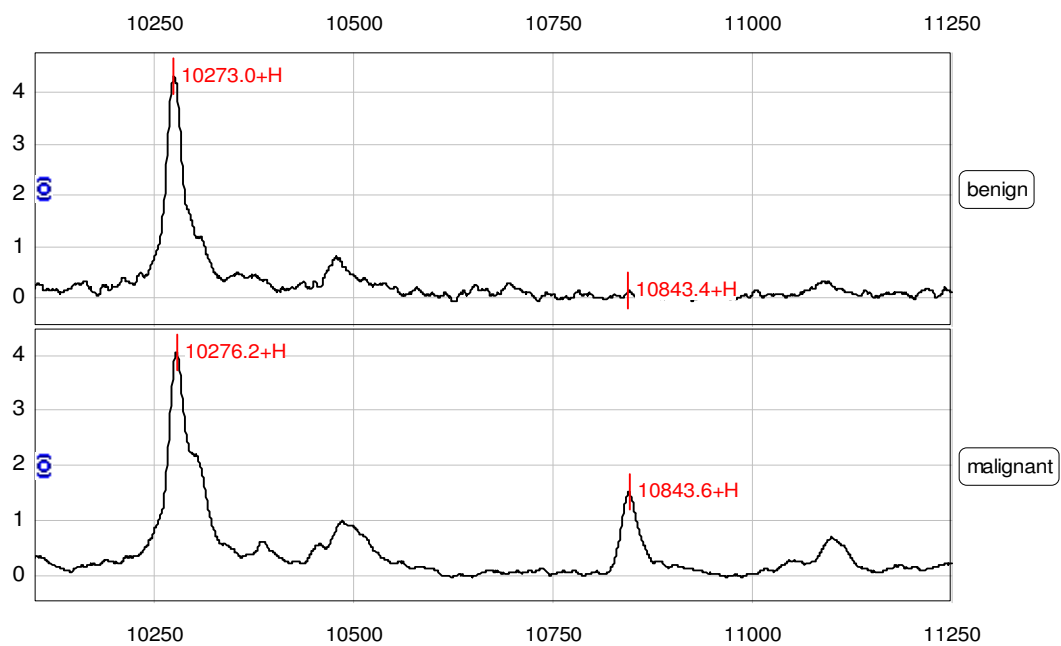

Supplement: Additional file 2 — Representative SELDI spectra. Spectra (x-axis: m/z ratio (Da); y-axis: normalized intensity) showing a differentially expressed peak at 10,884 Da (see Table 1 in the main text) between a patient with a benign and a patient with a malignant ovarian mass. The spectra were obtained using serum on a CM10 array. [file 1477-5956-10-45-S2.pdf]
